# Supporting Caregivers of Older Adults Through Education in Mindful Gratitude Practice

**DOI:** 10.1093/geroni/igaa057.053

**Published:** 2020-12-16

**Authors:** Sungsim Lee

**Affiliations:** Loyola University Chicago, Chicago, Illinois, United States

## Abstract

This presentation describes a supportive mindfulness practice for caregivers of older adults based on the principles of Won Buddhism (an integrative, a modernized Buddhism). As the aging population grows, there is a significant increase in recognition of the negative impact of caregiver stress on older adults’ quality of life. The ability for caregivers to deal compassionately with stress is essential, as caring for older adults can awaken feelings about one’s own vulnerability and mortality. The ‘Mindful Gratitude Practice’ offers a way to cope with stress, cultivate self-care, and improve the care of others. Relevant research will be summarized, which shows mindfulness and gratitude practice respectively benefit positive influence in both physical and emotional well-being. Mindful Gratitude Practice as a spiritual approach that fosters caregivers' emotional stability, reduces their stress and improves the relationship between older adults and their caregivers. In this presentation, three processes of Mindful Gratitude Practice will be described: 1. Understanding a mindfulness practice by establishing intention, attention, and attitude, 2. Learning the principles of a gratitude practice and implementation, and 3. Incorporating mindfulness into a gratitude practice. Research results have demonstrated that through this learning process, caregivers have acquired the concept of interconnectedness, experience grateful moments, and a deep feeling of appreciation in their caregiving relationships. The presenter will guide participants in a short experience of Mindfulness Gratitude Practice. Further readings and resources will be provided for those who are interested.

